# Protocol for the co-design of an online support service for adults with hearing loss

**DOI:** 10.1371/journal.pone.0310521

**Published:** 2024-09-26

**Authors:** Alicia Zou, Diana Tang, Melanie Ferguson, Kerry Sherman, Catherine McMahon, Liz Gill, Annie Lau, Jane Lee, Steve Williamson, Elizabeth Davies, Kate Sheng, Simon O’Toole, Andrew Georgiou, Payal Mukherjee, Peter Wolnizer, Bamini Gopinath

**Affiliations:** 1 Macquarie University Hearing Research Centre, Faculty of Medicine, Health and Human Sciences, Macquarie University, Sydney, NSW, Australia; 2 School of Allied Health, Curtin University, Perth, WA, Australia; 3 Lifespan Health and Wellbeing Research Centre, School of Psychological Sciences, Macquarie University, Sydney, Australia; 4 John Walsh Centre for Rehabilitation Research, Northern Sydney Local Health District, Sydney, NSW, Australia; 5 Kolling Institute, Faculty of Medicine and Health, The University of Sydney, Sydney, NSW, Australia; 6 Centre for Health Informatics, Australian Institute of Health Innovation, Macquarie University Sydney, Sydney, NSW, Australia; 7 Deafness Forum Australia, Canberra, ACT, Australia; 8 Australian Astronomical Optics, Macquarie University, Sydney, NSW, Australia; 9 Centre for Health Systems and Safety Research, Australian Institute of Health Innovation, Faculty of Medicine, Health and Human Sciences, Macquarie University, Sydney, NSW, Australia; 10 Faculty of Medicine and Health, Central Clinical School, The University of Sydney, Sydney, NSW, Australia; 11 Department of Medicine, Health and Human Sciences, Department of Clinical Medicine, Macquarie University, Sydney, NSW, Australia; 12 University of Sydney Business School, University of Sydney, Sydney, NSW, Australia; Public Library of Science, UNITED KINGDOM OF GREAT BRITAIN AND NORTHERN IRELAND

## Abstract

**Introduction:**

Untreated hearing loss is reported to negatively impact on an individual’s quality of life, affecting their psychological and physical health and placing them at greater risk of developing dementia. Despite this, hearing loss management is often delayed by up to a decade. This is likely due to difficulties in navigating the hearing care pathway, and the absence of a central, unbiased reference point for consumer-friendly hearing health information and resources. We intend to co-design an online support service for adults with hearing loss with the following aims: 1) to understand unmet needs and consumer barriers to accessing hearing health information, 2) to identify solutions to these unmet needs that can be developed into prototype ideas, 3) to incrementally build on iterations of a prototype until a usable online support service is developed and ready for real-life testing with end-users, and 4) to test and evaluate the usability, accessibility, and effectiveness of the prototype from the consumer’s perspective, so that the prototype can be refined into the final product.

**Methods and analysis:**

This will be a mixed method study. Consumers will be involved in all stages of the design of the project following the Hasso Plattner model of design thinking. The qualitative component will involve sprints and semi-structured interviews to access the consumer perspective and understand unmet needs and challenges regarding the access of online hearing health information. For the quantitative component, an online survey will be administered prior to prototype testing as part of the remote usability study to collect self-efficacy and eHealth literacy outcome measures via validated questionnaires. Data collection will also be performed post-prototype testing for evaluation purposes. Finally, heuristic evaluation of the prototype will be conducted by an eLearning expert to help refine the prototype into the final product.

## Introduction

Hearing loss has been identified as the third leading cause for years lived with a disability [1]. The ramifications of untreated hearing loss are profound and include communication difficulties and diminished social interaction [2–4]. Major flow-on health impacts associated with unaddressed hearing loss also include an increased risk of falls, depression, and poorer quality of life [2, 3, 5–7]. Importantly, midlife hearing loss has been identified as the single largest potentially modifiable risk factor for a future dementia diagnosis [8]. Therefore, early identification and management of hearing loss is crucial to mitigate these negative consequences [9]. In this context, targeted initiatives that provide adequate support and increase the uptake of interventions to treat hearing loss can offer significant value to adults with hearing loss, as well as to the broader health system and economy [8, 10].

However, for consumers, navigating hearing health pathways can be challenging. They must consider factors such as time, expense, and the extent of follow-up care to which they are willing to commit. Moreover, they are required to make complex decisions about the extensive range of hearing interventions, such as hearing aids and other devices, communication strategies, and aural rehabilitation, which can prove overwhelming and frustrating [11, 12]. As with most chronic diseases, timely and effective management of hearing loss is critical for improved health outcomes, which can be achieved through high health literacy and health self-efficacy [13]. Unfortunately, Australia currently lacks an authoritative, unbiased central reference point from which consumers can seek information on their hearing health.

To fulfil this unmet need, we propose the development of an innovative, Australia-first online support service for adults with hearing loss that will: a) provide access to credible, evidence-based and consumer-friendly educational materials, resources, and tools on hearing loss; b) facilitate improved life-course perspective of individual hearing health needs and risk factors; c) promote healthy hearing behaviours through online self-assessment and tailored care plans. This novel online support service promises to provide millions of adults with hearing loss with access to digital evidence-based information and practical resources on hearing health. The goal of this study is to improve quality of life by enabling adults with hearing loss to manage their hearing loss by increasing health literacy and self-efficacy of hearing loss management through access of this purpose-built digital service.

This study’s aims are four-fold:

1. To better understand unmet needs and challenges in accessing and engaging hearing health information and resources for adults with hearing loss.
2. To conduct a series of ideation meetings to identify solutions that can be developed into prototype ideas to solve the unmet needs of consumers with hearing loss.
3. To incrementally build on previous iterations until a usable, testable prototype is developed and ready for real-life testing with end-users.
4. To test and evaluate the usability, accessibility, and effectiveness of the prototype from the consumer’s perspective, and then use these evaluation data to further refine and propel the prototype into the final draft.

## Materials and methods

This study was approved by the Macquarie University Human Research Ethics Committee (ID:16744) on the 20th June 2024.

An advisory group will be established at the outset of this study and will remain involved throughout its duration. The advisory group will guide study governance, design, data collection and analysis, and the dissemination of findings. This group will include hearing health representatives from academia and industry bodies, ten representatives from Deafness Forum Australia member organisations, and ten Community and Consumer Involvement Panel (CCIP) members with lived experience of hearing loss. CCIP members will be recruited internally through Deafness Forum Australia networks. Interested participants will be screened for eligibility (over 18 years old; have a clinician-verified hearing loss; and live in Australia) and asked to confirm their understanding of the ongoing responsibilities involved with participation. Due diligence will be observed to ensure diversity among the CCIP regarding gender, age, ethnicity, and geographical living classification. The ten Deafness Forum representatives will also be recruited internally from their 45 member organisations. Recruitment for CCIP and Deafness Forum representatives will take place between the 5^th^ of July 2024 and the 31^st^ of October 2024. Eligible consumers and Deafness Forum representatives will be asked to provide informed written consent by signing and returning a Participant Information and Consent Form (PICF) detailing the responsibilities of advisory group members. CCIP members will be compensated from $46.39/hr (based on Health Consumers NSW renumeration and reimbursement guidelines) for their involvement in the study, which may include sprints, interviews, meetings, and travel time.

**Fig 1:**
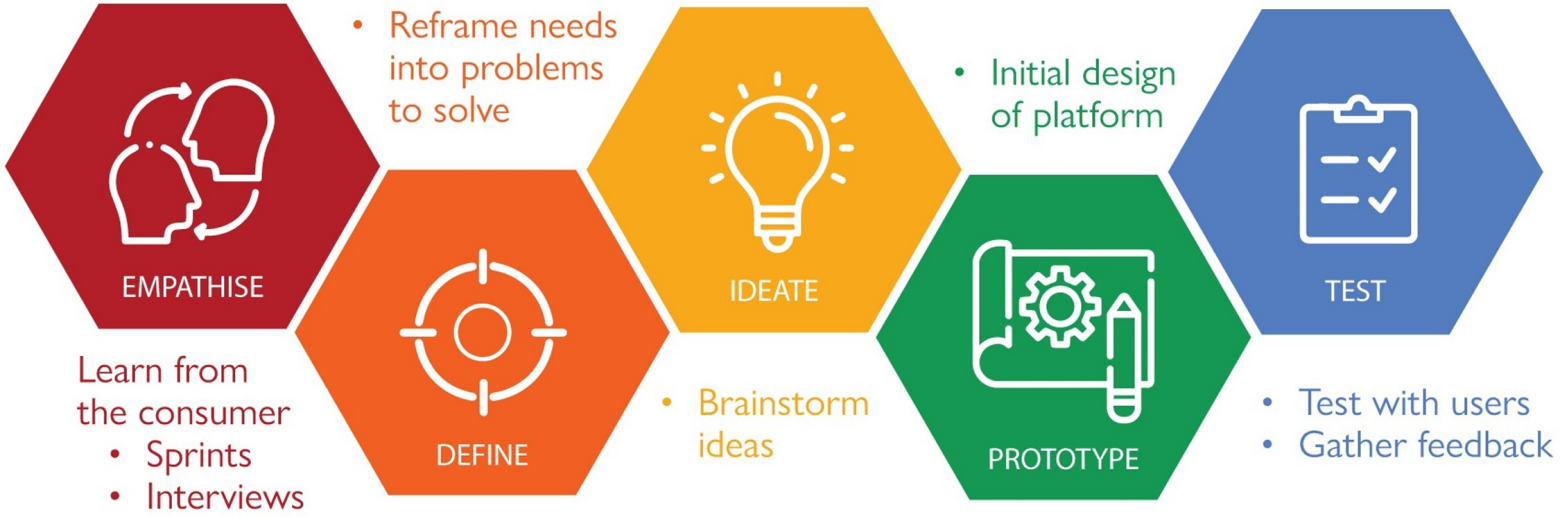
Hasso Plattner model of design thinking. The Hasso Plattner model of design thinking places consumers at the centre of each of the five phases of design thinking: Empathise, Define, Ideate, Prototype, and Test.

Qualitative and quantitative data will be collected for this mixed-method, observational study. The study will be conducted following the Hasso Plattner model of design thinking, which specifies that consumers should be placed at the centre of all five phases of design thinking: Empathise, Define, Ideate, Prototype, and Test [14] (Fig 1).

### Phase 1: Empathise and define

To design an effective and consumer-friendly online support service for adults with hearing loss, it is necessary to understand the unmet needs and challenges of consumer access to hearing health information and resources. Phase 1, ‘Empathise and Define’, will begin with two-hour ‘Sprint’ sessions (Fig 1), typically run over a short, fixed period, allowing for intense focus and rapid progress. These sessions will enable the project team to gain a better understanding of the barriers and facilitators to accessing and using online support services for hearing loss. Modern user experience design methodologies relevant to the digital space will be incorporated into these sessions to ensure that problems with existing solutions are adequately captured and can be alleviated by the online support service. The ten CCIP members will be invited to share and discuss their experiences with hearing loss to help inform the design of the digital service. Prior qualitative research involving adults with hearing loss indicates that 10–14 participants will likely be required to reach data saturation [15]. Data gathered from these sessions will be clarified and refined in follow-up semi-structured interviews with CCIP members. Interview guides will be structured around the Capability, Opportunity, Motivation-Behaviour (COM-B) model of behaviour change, which suggests that, in order for a behaviour to be changed, one or more of these three interconnected components must be influenced [16]. Therefore, interview questions will be mapped to these three components, which will enable the identification of barriers and facilitators to the use of an online support service so that one might design solutions for its improved application and use. Interviews will be recorded, de-identified, and professionally transcribed using NVivo, a qualitative data analysis software. Transcripts will be manually checked against the audio recording for accuracy. Participants will be given two weeks to review and edit their transcripts. Thematic template analysis of the transcripts will then be performed under the Theoretical Domains Framework, allowing the data to be rigorously organised and coded [17, 18].

### Phase 2: Ideate

With the advisory group, the research team will review the data obtained during the ‘Empathise and Define’ phase and reframe the unmet needs of consumers and stakeholders as problems to solve by formulating “how might we” statements (Fig 1). The group will partake in a series of ideation meetings following a “diverge-converge-vote” structure as proposed by IDEO, an organisation specialising in human-centered design innovation. Advisory group members will diverge into groups to brainstorm solutions to the issues identified before converging to discuss and cluster ideas to be voted upon. Solutions that receive the majority vote will be prioritised in the prototype online support service.

Once the solutions to the consumer-identified problems have been decided, a literature review will be conducted by the research team to identify applicable theories (e.g. Health Action Process Approach, Bagozzi’s Goal Theory) to guide the design of the prototype. Interdisciplinary databases (e.g. PubMed) will be searched for peer-reviewed English-language articles using Medical Subject Headings and their variants (e.g. theory or model, intervention or program), and the most suitable theories will be determined by the research team. This theory-based design process allows for relevant constructs to be mapped to consumer needs so that the corresponding intervention techniques can be created and built into the platform.

### Phase 3: Prototype

As the online support service is intrinsically an evidence-based intervention, the design of the prototype will be guided by the Intervention Mapping approach, which combines theoretical and empirical evidence to propose effective real world intervention techniques [19]. Intervention Mapping is a systematic framework used to develop theory-based health promotion programs, as it considers population needs to intervene at individual, interpersonal, organisation, and community levels [20].

Theories identified during the Ideate phase will be mapped to corresponding evidence-based intervention techniques and translated into a series of minimum viable products (MVPs) to be tested in the prototype. MVPs will be built following a predetermined set of criteria based on Persuasive Systems Design, to influence a consumer behaviour change towards better healthcare management though the use of specific design features [21]. These features may include client-tailored information, interactivity (including social support), presentation of content, credibility, and multimedia graphics. An example of an MVP that might be considered in the development of this prototype are Reusable Learning Objects (RLOs), or short multimedia interactive videos. RLOs have demonstrated promise as valuable tools and educational supports for hearing loss management [22, 23]. MVPs may also consist of a series of connected features that can be used to validate a product or idea. MVP creation will follow an iterative build-measure-learn cycle, where each version will be evaluated by the study’s advisory group for its effectiveness in addressing end-user needs. Feedback from the advisory group will help determine if MVPs should be kept for the next version of the prototype or modified to test another assumption. Over 12–18 weeks, MVPs will be incrementally built upon previous iterations until a functional, testable prototype of the online platform is developed.

Prior to user testing, the research team will set a go/no-go criterion for the prototype based on whether a proposed solution achieves a 70–80% consensus (among the team) that it would provide value to the end-user. This arbitrary threshold attempts to balance the inclusion of information materials and tools that consumers want and that are effective with what researchers and stakeholders perceive to be feasible and beneficial. If consensus is achieved, the prototype will proceed on to user testing.

### Phase 4: Test

The prototype will be made available to users for testing during this phase and will involve three components: a think-aloud test, a remote usability study, and the heuristic evaluation. Recruitment for the Test phase will likely take place between the 1^st^ of May 2026 and the 31^st^ of October 2026.

The eligibility criteria of participants for both think-aloud and remote usability studies are individuals aged 18 and over who have a hearing loss, are fluent in English, and have regular access to and can use usual internet communications (e.g. browsing websites and/or social media platforms). The remote usability study also requires participants to have access to a compatible web browsing device, such as a laptop. Participant recruitment will be conducted through Deafness Forum Australia channels and investigator networks. Interested parties will be contacted by the project coordinator online or via telephone to screen for their eligibility and to verify their commitment to participating. Consumers who satisfy these conditions will be asked to review and complete the PICF via Research Electronic Data Capture (REDCap) to confirm and provide informed written consent to their participation. Enrolment into think-aloud and remote usability studies will occur on a “first come, first served” basis until the recruitment target for each study is achieved. According to principles decided by experts in the field, as well as prior usability studies of a similar nature, a sample size of 10 participants will be required for the think-aloud tests, while 30 will be needed to complete the remote usability study [24]. Upon receipt of signed consent, the project coordinator will schedule all appointments for the think-aloud test until its completion by all recruited participants. Remote access to the prototype will then be arranged to conduct the remote usability study. All participants will be compensated with a $30 GiftPay voucher once they have completed their section of the study.

Think-aloud test sessions are commonly used to evaluate the usability of digital systems and services [25]. This will entail an in-person one-hour test session of the prototype and all its available functions. Participants will be guided by the project coordinator to log into and browse the prototype on a desktop computer. All actions performed by these participants on the prototype will be recorded automatically and non-intrusively. Participants will also be audio-recorded during these think-aloud sessions to capture user experiences whilst using the prototype. Transcripts of the sessions will be analysed to document general perceptions of the prototype and to pinpoint the key strengths and weaknesses identified by participants. Using the content analysis method, transcripts will be read to identify codes and reread to discern higher level themes. Codes and themes will be interpreted following the COM-B system of health behaviours and the Theoretical Domains Framework to inform the refinement of the prototype.

For the remote usability study, participants will be asked to enrol online through the project website to access the prototype. Each participant will be provided with an individualised login, which can be activated with a self-chosen password to access the prototype. On their personal devices, participants will have 24/7 remote access to the prototype and all its available content over a two-week period. The project coordinator will provide instructions on and encouragement around the use of interactive forums on the online service prototype, which will be regularly monitored by the project coordinator during this time (e.g. to ensure that no inappropriate content is being posted). Google Analytics will be embedded within the digital platform to collect web analytics from participant use, including number of logins, pages visited, and posts made. Participants will also be required to complete an online evaluation survey prior to prototype testing, which will collect demographic and hearing loss information, as well as responses to validated questionnaires including the Attitudes Towards Loss of Hearing Questionnaire, eHealth Literacy Scale, and the Self-Efficacy to Manage Chronic Disease Scale [26–31] (Table 1). These validated questionnaires will be re-administered immediately post-prototype testing to assess the impact of the online support service on these outcome measures. The post-prototype survey will be supplemented by the validated Systems Usability Scale questionnaire, along with questions to gauge consumer perceptions of the prototype in terms of usefulness, helpfulness, and acceptability on 5-point Likert scales ranging from very helpful to very unhelpful. Free text entry spaces will allow participants to give reasons for their rating and general feedback. Data from the online evaluation surveys will be stored in REDCap.

**Table 1:** Usability and health outcome measures collected in the Test phase.

Validated questionnaire	Description
**System Usability Scale (SUS) [26]**	10 questions that contain a mix of positive and negative items pertaining to different usability aspects of web applications e.g. effectiveness, efficiency, and satisfaction. Total scores range between 0–100 and scoring ≥68 is regarded as above average in terms of usability quality of the intervention.
**Self-Efficacy to Manage Chronic Disease Scale [28]**	Validated scale made up of six items on a visual-analog scale, ranging from 1 (not at all confident) to 10 (totally confident). The score is the mean of the items, with the score range 1–10. A higher score indicates higher self-efficacy or more confidence in managing a chronic disease (hearing loss).
**eHealth Literacy Scale [29]**	Validated 8-item measure of eHealth literacy measuring consumers’ combined knowledge, comfort, and perceived skills at finding, evaluating, and applying electronic health information to health problems. Scores greater than and equal to the median value is classified as high eHealth literacy level.
**Attitudes Towards Loss of Hearing Questionnaire [30]**	Validated 22-item scale that evaluates denial of hearing loss, negative associations, negative coping strategies, manual dexterity and vision, and hearing-related esteem. Each item is scored on a 5-point Likert scale indicating level of agreement (strongly disagree, neither agree nor disagree, to strongly agree).

Finally, heuristic evaluation will be used to help refine the prototype into the final online support service. An eLearning expert, not involved in the prototype build, will evaluate the intervention according to Nielsen’s 10 heuristics, which is recognised as the benchmark for good interface design [32]. This set of essential rules will assess the prototype in areas such as user control and freedom, consistency, error prevention, and aesthetic design. An overall score will be calculated, combining a three-level rating measuring the frequency a usability problem occurs (1 = only in one place to 3 = as part of the main persistent navigation interface) with five-point ratings for its severity (0 = no usability problem to 4 = usability catastrophe) [32]. Scores range from 0–12, with higher scores indicating a more severe and widespread usability problem.

### Statistical analysis

Descriptive statistics will be used to summarise the dataset and provide central tendency statistics. Means and standard deviations or medians and interquartile ranges will be used to summarise continuous outcomes, such as usability scores. Chi-squared or Fisher’s exact tests will be used to compare binary outcomes while independent two sample t-tests will be used for continuous outcomes. A general linear model will be fitted to investigate differences in, for example, self-efficacy and eHealth literacy scores pre- and post-prototype testing. Potential confounders will be adjusted for, and subgroup analyses (age and sex) will be performed. Visual investigation (e.g. scatter plots) and logistic regression analysis of outcome measures will also be conducted to determine whether unique consumer characteristics can predict thresholds of use of individual components of the prototype, for example, accessing interactive tools and information resources.

### Refinement and scale-up

The prototype will be refined following the Test phase based on qualitative feedback and quantitative evaluation by end-users and the eLearning expert. An agile development process will be initiated between the advisory group, web application developers, and consumers not involved in previous design steps, who will engage in bi-weekly sprint meetings until the prototype is refined into the final draft [33]. The research team will then work with Macquarie University, Deafness Forum, and Google to develop a marketing strategy to promote this novel online support service on a wider scale.

## Discussion

Currently, consumers must take it upon themselves to navigate the complex hearing care pathway without adequate guidance or access to a centralised unbiased source of hearing health information, to inform their choices regarding rehabilitation and interventions. This likely contributes to the 8.9-year average delay of adults seeking help for their hearing loss after noticing a decline [34]. However, it is recognised that earlier and better management of chronic diseases such as hearing loss results in better health outcomes [13]. In particular, consumers who are actively engaged in their health care, through health services and information delivered or enhanced through the internet, have improved knowledge, self-management behaviours, and self-efficacy when it comes to managing their disease [35]. We hope to empower consumers to better manage their hearing loss and encourage the uptake of relevant interventions through access to our online support service to prevent poor quality of life, future risk of depression, and cognitive decline [11].

The potential risks of participating in this study are minimal. All participation is voluntary, and participants will be able to withdraw from the study at any time without given reasons. Withdrawal will not impact participant relationships with organisations involved in the study.

A one-page summary of the findings from this study will be distributed to study participants via post or email, if they have chosen to receive this information. Findings will also be disseminated through publications in peer-reviewed journals, presentations at conferences, and partner institution networks. All findings will be de-identified prior to their release.
